# The appearances of a parrot’s peak and petal rims in brucellar spondylitis

**DOI:** 10.1590/0037-8682-0056-2025

**Published:** 2025-03-31

**Authors:** Li-jie Guo, Yong-Sheng Yu, Yi Zhang

**Affiliations:** 1 Shanghai Sixth People’s Hospital Affiliated to Shanghai Jiao Tong University School of Medicine, Department of Infectious Diseases, Shanghai, China.

**FIGURE 1:**
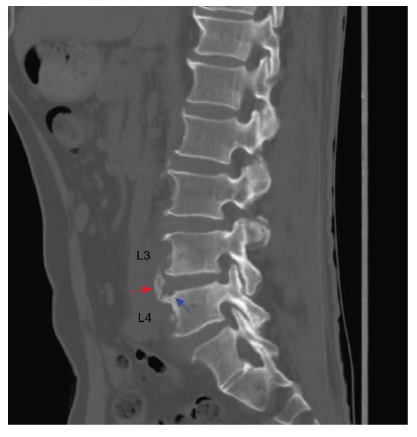
Computed tomography scan (sagittal section) showing focal erosion at the anterior superior corner (blue arrow) (known as Pedro Pons’ sign) of the L4 vertebra with prominent osteosclerosis and osteophytes (red arrow) resembling “a parrot’s beak”.

**FIGURE 2:**
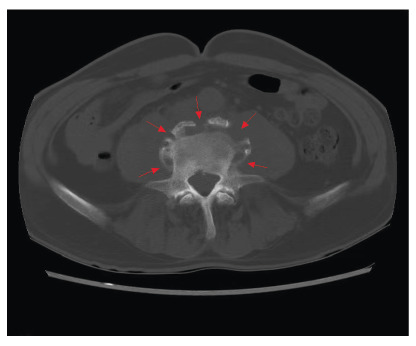
Computed tomography scan (transverse section) showing apparent destruction around the edge of the L4 vertebral body with prominent osteosclerosis and osteophytes (red arrows) resembling “petal rims”.

**FIGURE 3:**
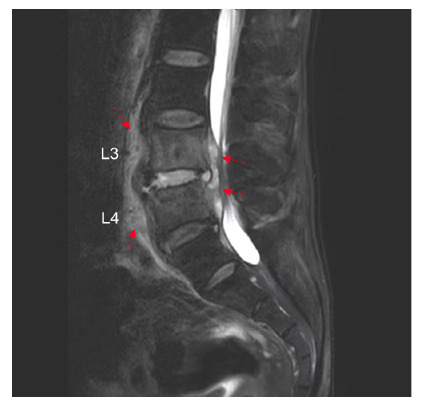
Magnetic resonance imaging (sagittal section) showing abnormal signals in the L3 and L4 vertebral bodies and the intervertebral disc accompanied by paravertebral and epidural abscesses (red arrows).

A 66-year-old man with a history of contact with cows presented with intermittent fever, sweating, progressive lower back pain, and limited range of motion persisting for 3 months. Physical examination revealed percussion pain in the L3 and L4 vertebrae. The white blood cell count was 7.0×10^9^/L, with 74.4% neutrophils and 17.6% lymphocytes. The inflammatory marker levels were elevated, with erythrocyte sedimentation rate of 71 mm/h and C-reactive protein level of 69 mg/L. Computed tomography (CT) scan revealed focal erosion at the anterior superior corner (termed Pedro Pons’ sign^1^) of the L4 vertebra with prominent osteosclerosis and osteophytes resembling “a parrot’s beak” (Figure 1), and apparent destruction around the edge of the L4 vertebral body with prominent osteosclerosis and osteophytes resembling “petal rims” (Figure 2). Magnetic resonance imaging revealed abnormal signals in the L3 and L4 vertebrae accompanied by paravertebral and epidural abscesses (Figure 3). The patient underwent CT-guided percutaneous drainage. *Brucella abortus* (*B. abortus*) was cultured from both blood and pus. The serum agglutination test was positive for *Brucella* with a titer of 1:640, confirming the diagnosis of brucellar spondylitis (BS). Antibiotic therapy was continued for 4 months. The patient responded satisfactorily to the medical treatment without any complications.

In addition to Pedro Pons’ sign, the appearances of a parrot’s peak and petal rims are described as the destructive vertebral lesions associated with osteosclerosis and osteophyte formation^1^ ^,^ ^2^. The presence of a combination of these lesions is a characteristic radiological feature of BS, which can help distinguish BS from tuberculous spondylitis^2^.
