# Erratum

**DOI:** 10.1590/0037-8682-0056e-2025

**Published:** 2025-05-09

**Revista da Sociedade Brasileira de Medicina Tropical/Journal of the Brazilian Society of Tropical Medicine**

Title: The appearances of a parrot’s peak and petal rims in brucellar spondylitis 

 Vol.:58 | (e02001-2025) | 2025 | doi: https://doi.org/10.1590/0037-8682-0056-2025

**Page 1/1**

 In addition to Pedro Pons’ sign, the appearances of a parrot’s peak and petal rims are described as the destructive vertebral lesions associated with osteosclerosis and osteophyte formation^1,2^. The presence of a combination of these lesions is a characteristic radiological feature of BS, which can help distinguish BS from tuberculous spondylitis^2^.

**Should read:**

 In addition to Pedro Pons’ sign, the appearances of a parrot’s **beak** and petal rims are described as the destructive vertebral lesions associated with osteosclerosis and osteophyte formation^1,2^. The presence of a combination of these lesions is a characteristic radiological feature of BS, which can help distinguish BS from tuberculous spondylitis^2^.

